# Toxoplasmosis Infection and Cognitive Deficit after Electroconvulsive Treatment (ECT), Is There a Connection?

**Published:** 2012

**Authors:** John E. Berg

**Affiliations:** Oslo and Akershus University College, Faculty of Health Sciences, Norway

**Keywords:** Electroconvulsive treatment (ECT), Toxoplasmosis, Cognitive deficit

## Abstract

Electroconvulsive treatment (ECT) has developed over 70 years to a modern, effective way of lifting depressive moods. Memory loss and visual acuity after electroconvulsive treatment is the only remaining relevant criticism of the treatment modality when considering the overall rate of remission from this treatment compared to all other treatment modalities. A depressive state impedes memory, and memory improves on several qualities of cognition after treatment. However, the comparison of a person’s memory ability from the months before depression started to the level after a course of ECT is never performed, for obvious reasons. Some infectious diseases are known to influence memory negatively through effects on the dopamine receptors. More specifically, former toxoplasmosis infection may be a factor. Preliminary data on titres of toxoplasma IgG may indicate a connection to the development of long-standing memory problems after ECT.

## INTRODUCTION

The question of memory loss after electroconvulsive treatment is unsettled and interferes with the scientific handling of severe depression. Lay concerns get a wide coverage in the media and in scientific journals [1]. However, thorough reviews of the literature and practice seem to not get the same coverage [2, 3]. Memory loss ((λήθη)=lethe), named after the river in the underworld in Greek mythology, has as its counterpoint truth (αλήθη) not lie, according to the Greek view. It has been previously hypothesised that meeting the truth may be a problem for the depressed after the often speedy recovery during ECT [4]. On the other hand, certain infections by protozoa and bacteria may also have an impact on central nervous function, even long after the index infection has subsided [5]. 

Lafferty discussed the cultural influence of Toxoplasma gondii infection on behaviour and stated: “The latent prevalence of a long-lived and common brain parasite, Toxoplasma gondii, explains a statistically significant portion of the variance in aggregate neuroticism among populations, as well as in the 'neurotic' cultural dimensions of sex roles. A link between culture and T. gondii hypothetically results from a behavioural manipulation that the parasite uses to increase its transmission to the next host in the life cycle: a cat. While latent toxoplasmosis is usually benign, the parasite's subtle effect on an individual personality appears to alter the aggregate personality at the population level. Drivers of the geographical variation in the prevalence of this parasite include the effects of climate on the persistence of infectious stages in soil, the cultural practices of food preparation and cats as pets. Some variation in culture, therefore, may ultimately be related to how climate affects the distribution of T. gondii, though the results only explain a fraction of the variation in two of the four cultural dimensions, suggesting that if T. gondii does influence human culture, it is only one among many factors” [6]. 

In a Russian study from 2001, Lobzin et al. asserted that patients with chronic toxoplasmosis had “marked disorders in higher nervous activity and psychomotor system” [7]. They found many cases among these patients with mistakes in attention tests, in head calculating and with delayed memory at a degree impeding functioning, in occupational tasks as technical operators. This is also corroborated in a Polish case report showing memory and language impairment after toxoplasmosis infection [5]. The patient had mild cognitive decline with apraxia and difficulties with attention even several months after the infection. 

In his millennial article “Memory loss after electroconvulsive treatment during its 70 years of practice”, Max Fink stated that, after the period with unmodified ECT, when fractures and panic were the great obstacles to accepting ECT, memory changes took their place [8]. The use of general anaesthesia and muscle relaxants made the procedure calm and more acceptable to patients and staff alike. The objections to the treatment from patients and advocates of the anti-psychiatry movement have been rooted in the old way of performing the procedure [1]. 

Individual patients do feel uncomfortable with the, albeit short, procedure of anaesthesia, and some are frightened that they would not wake up after the session. 

A multitude of psychometric tests exist that were constructed to tap memory changes in patients [9]. The Sackeim group in New York used the following tests in patients undergoing ECT: the Cognitive Failures Questionnaire (CFQ), the Squire Memory Complaint Questionnaire (SMCQ) and the Global Self-Evaluation of Memory (GSE-My). They appear to give disparate results, as shown by Berman and also Brakemeier [10]. 

Fraser et al. studied the effect of ECT on autobiographical memory from 15 suitable studies [11]. She found that autobiographical memory impairment occurs as a result of ECT, but the memory loss was short term (<6 months) with objective measures, whereas the amnesia was judged more long term with subjective accounts. Fraser recommended further studies that would focus on the separate impact of the depression itself on memory. King et al. [12] performed this kind of research. Autobiographical memory is related to the depressive disorder itself and to other factors, such as comorbidity, trauma, age, ruminative state, culture and executive function, and is also related to ECT. The impact of each factor in patients is unknown, but is clearly present. The testing of autobiographical memory should be aimed at differentiating episodic from semantic memory and time from emotion. 

In another study, Meeter et al suggested “it is possible that before ECT, patients could not recall news events due to depression. After ECT, the recall of some memories was improved, but other memories were impacted by ECT and could thus not be recalled or recognised” [13]. Recognition tests of remote memory have been employed in many previous studies. After employing further tests, Meeter et al. concluded by stating that ECT, as now practised, does not cause significant lasting retrograde amnesia. 

A difference between aspects of memory deficits has been observed when comparing patients referred for ECT and not referred [14]. The ECT patients showed more executive function deficit, but less visuospatial memory reduction than equally severely depressive patients not referred for ECT. 

The measurement of memory loss to prove any connection to ECT is phenomenologically and methodologically difficult [15-18]. There is seldom access to measurements of memory function in patients from the time before they developed the depression. However, it may be possible to access information on the level of parasites and protozoa in the society [6, 19]. Measuring memory shortly before an ECT series starts will always show a degree of memory impairment as the depression itself reduces memory acuity. 

The existence of a common biological factor that might fluctuate between cultures would complicate the evaluation of memory loss after ECT. Tests have been initiated to evaluate this connection regarding former toxoplasmosis infection in ECT patients. 

## HYPOTHESES

The memory loss seen in some patients undergoing electroconvulsive treatment (ECT) is not explained by the treatment alone. A growing body of evidence suggests that the parasite-entrained dysregulation of dopamine metabolism may play a role. This dysregulation may lead to memory impairment in patients not otherwise prone to memory problems. Laboratory data was collected on toxoplasmosis infection status for seven patients who underwent a series of electroconvulsive treatments. None had an earlier recognised cognitive deficit or memory loss. The indication for ECT was either a unipolar or a bipolar depressive episode. Memory loss was defined as forgetting places, names and appointments more than two months after the end of the ECT series. The patients were observed during ECT sessions and at least six months after the end of the ECT series. 

## RESULTS

Three of the seven patients investigated suffered from memory loss more than two months after the end of the ECT series (see table 1). Two of the three had an elevated titre of toxoplasmosis IgG, but none of the four patients without memory loss had an elevated value of toxoplasmosis IgG. All patients were of a similar age, ranging from 46 to 63 years, and included three men and four women. 

**Table 1:** Toxoplasmosis IgG and IgM titre in patients after ECT and development of post treatment cognitive deficits

**Case no. **	**Age**	**Sex**	**Toxoplasma IgG**	**Toxoplasma IgM**	**Cognitive deficit as post ECT memory loss**
1	54	F	0.0 IU/ml	Negative	No
2	46	M	0.3 IU/ml	Positive, low titre	No
3	53	F	171.1 IU/ml	Negative	**Yes**
4	40	F	0.1 IU/ml	Negative	No
5	63	M	0.2 IU/ml	Negative	No
6	49	M	0.2 IU/ml	Negative	**Yes**
7	47	F	13.9 IU/ml	Negative	**Yes**

## DISCUSSION

The observations of this preliminary study of toxoplasmosis titre in patients undergoing ECT may give some support to the findings of changes in mental capacity and memory shown by others [20]. The mechanism may be through the effect of Toxoplasma gondii on mammalian dopaminergic cells. The K+-induced release of dopamine increases several-fold with a direct correlation between the number of infected cells and the quantity of dopamine released. When a toxoplasma infection deteriorates it may lead to encephalitis causing gradual forgetfulness and other mental and infection-related symptoms as reported in a review and case study by Habek et al. [21]. The case presented with a two-month history of cognitive decline. The most prominent deficit was in short-term verbal and visual memory and recognition, which was very similar to the symptoms presented by case 6. The IgG titres in the nine cases described by Habek et al. were all much higher, as expected. The degree of behaviour changes after toxoplasma infection appear to be parasite strain-dependent [22].

There are two different manipulations possible during ECT to reduce the risk of memory problems after ECT [23, 24]: 

•Ultra-brief stimulation at 0.25 or 0.30 msec

•Right unilateral stimulation [25]

These changes may be applied in a well-equipped ECT suite. Thus, the clinicians would have methods available to minimise the risk of cognitive side-effects. The anti-depressive effect of ECT using these alternative manipulations is, however, reduced according to some researchers in the field [15, 26, 27].

## DISCLOSURE

The authors report no conflicts of interest in this work.
